# Beyond Acute Infection: A Conceptual Framework Linking Zoonotic Bacterial Pathogens to Pulmonary Fibrosis and Lung Carcinogenesis

**DOI:** 10.3390/biomedicines14081776

**Published:** 2026-08-06

**Authors:** Ju Hee Lee, Nam Yee Kim, Chang-Min Choi, Minjeong Yeon

**Affiliations:** 1 Department of Pulmonary and Critical Care Medicine, Asan Medical Center, University of Ulsan College of Medicine, Seoul 05505, Republic of Korea; midory4597@gmail.com (J.H.L.); ccm@amc.seoul.kr (C.-M.C.); 2 Division of Vector-Borne Diseases, Department of Diseases Research, Incheon Metropolitan City Institute of Public Health and Environment, Incheon 22320, Republic of Korea; newny33@gmail.com; 3 Department of Biochemistry, Chungbuk National University College of Medicine, Cheongju 28160, Republic of Korea

**Keywords:** zoonotic infections, pulmonary fibrosis, lung carcinogenesis, chronic inflammation

## Abstract

Zoonotic bacterial pathogens are transmitted through various routes and are traditionally associated with acute febrile illnesses that may include pulmonary complications. However, in some survivors, the disease extends beyond the acute phase, leading to the remodeling of pulmonary architecture and driving progressive fibrosis. Although no direct cases have been reported, these pathogens may plausibly predispose injured lungs to carcinogenesis, similar to the well-recognized phenomenon of tuberculosis-associated scar cancer. As such long-term sequelae remain largely overlooked in current clinical practice, their potential contribution to fibrotic and malignant lung disease represents a critical and underexplored knowledge gap. This review proposes a unified mechanistic framework linking acute pathogen-mediated alveolar damage to chronic pulmonary fibrosis and subsequent lung carcinogenesis. We delineate four convergent biological pillars driving this continuum: (1) pathogen persistence establishing chronic Interleukin-1β (IL-1β)/Tumor necrosis factor-α (TNF-α)-mediated inflammation; (2) sustained TGF-β signaling and mechanotransduction driving progressive extracellular matrix remodeling; (3) unresolved reactive oxygen species (ROS) generation causing profound oxidative DNA damage; and (4) aberrant epithelial–mesenchymal transition (EMT) that perpetuates fibrosis and generates pre-malignant cell populations. Together, these sequelae alter lung biomechanics, suppress local immune surveillance, and create a mutagenic environment that is highly conducive to malignant transformation. Although direct epidemiological data is still emerging, the significant mechanistic overlap with idiopathic pulmonary fibrosis (IPF) presents a compelling rationale for shared oncogenic risk. We advocate for a paradigm shift in clinical practice, emphasizing the potential value of long-term surveillance for survivors of severe pulmonary infections. By integrating infectious diseases, pulmonology, and oncology, this framework highlights a neglected cause of fibrotic lung disease and establishes a foundation for future translational research.

## 1. Introduction

Zoonotic pathogens are responsible for many emerging infectious diseases worldwide, accounting for over 60% of all documented infectious disease events [1]. Zoonotic bacterial pathogens including *Coxiella burnetii*, *Leptospira* spp., *Rickettsia* spp., and *Brucella* spp. are transmitted through diverse routes and often utilize multiple transmission mechanisms (Table 1) [2,3]. These four bacterial pathogens are well-recognized zoonotic bacteria worldwide and provoke pulmonary complications and potentially long-term inflammatory changes. Among the diverse clinical manifestations of these infections, pulmonary involvement is an often-overlooked complication. Although bacterial zoonotic infections are traditionally regarded as acute febrile illnesses, they can persist or re-emerge even after apparent clinical control [4,5,6]. In some cases, they have been associated with persistent or interstitial lung changes that extend well beyond the acute phase [7,8]. Although the relationship between zoonotic infection-mediated pulmonary injury and lung cancer has not yet been reported, the concept that pulmonary scarring may predispose to lung cancer has been proposed in other contexts. Tuberculosis-associated scar cancer is a well-recognized clinical example in which post-inflammatory fibrotic scars are epidemiologically associated with increased lung cancer risk, particularly adenocarcinoma and squamous cell carcinoma [9,10]. This established paradigm supports the broader hypothesis that chronic infection-driven fibrotic remodeling, regardless of microbial etiology, may create a permissive microenvironment for malignant transformation. In this narrative review, we present a conceptual framework that connects zoonotic bacterial infections, pulmonary fibrosis, and potential lung carcinogenesis. Additionally, we explore the shared mechanisms that may drive this pathological progression and identify key knowledge gaps to guide future mechanistic and epidemiological research.

## 2. Materials and Methods

### 2.1. Literature Search Strategy

An extensive literature search was conducted for this review using the PubMed and Google Scholar databases, covering publications from 1980 to June 2026. The following search terms were used, individually and in combination: “*Coxiella burnetii* pulmonary,” “Q fever pneumonia,” “*Leptospira* pulmonary hemorrhage,” “*Rickettsia* pneumonitis,” “*Brucella* pulmonary,” “zoonotic bacterial pneumonia,” “zoonotic transmission route,” “animal reservoir zoonosis,” “chronic pulmonary disease,” “idiopathic pulmonary fibrosis (IPF),” “pulmonary fibrosis pathogenesis,” “interstitial lung disease (ILD),” and “chronic infection pulmonary fibrosis.” Retrieved sources were included if they met the following criteria: (1) the source was a research article, systematic review, narrative review, case report, or conference abstract; (2) a digital object identifier (DOI) was available; and (3) the study reported findings from mouse or human subjects.

### 2.2. Generative AI Disclosure

**Figure 1:**
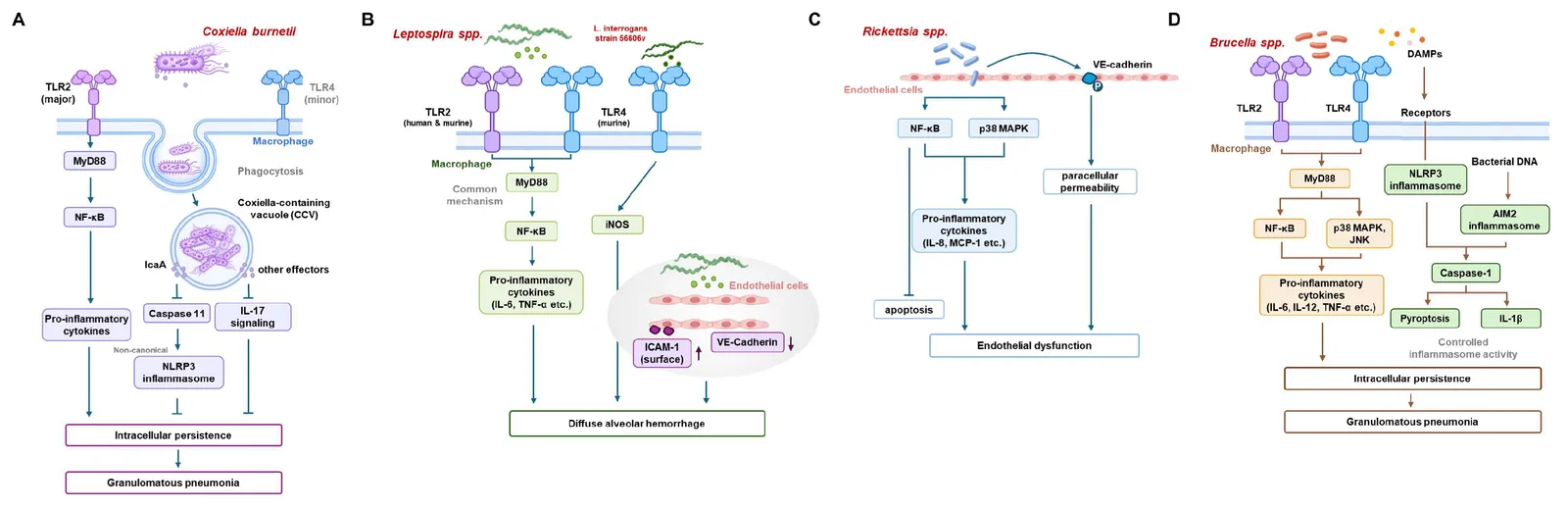
Pathogen-specific molecular mechanisms linking innate immune recognition to pulmonary injury. (**A**) *C. burnetii* is sensed by TLR2/TLR4 on macrophages, driving MyD88-NF-κB-dependent cytokine release. Separately, CCV-resident effectors suppress caspase-11/NLRP3 and IL-17 signaling, enabling intracellular persistence and granulomatous pneumonia. (**B**) *Leptospira* spp. signal through TLR2 (human/murine) or TLR4 (murine) on macrophages via MyD88-NF-κB. Strain 56606v exclusively induces iNOS via TLR4. Separately, endothelial cell infection induces ICAM-1 expression, reducing VE-cadherin expression. Both pathways drive diffuse alveolar hemorrhage. (**C**) *Rickettsia* spp. invade endothelial cells, activating NF-κB/p38 MAPK and VE-cadherin phosphorylation, together causing endothelial dysfunction. (**D**) *Brucella* spp. engage TLR2/TLR4 on macrophages, activating MyD88-NF-κB, p38 MAPK, JNK MAPK mediated pro-inflammatory cytokine release. In addition, both DAMPs and bacterial DNA trigger NLRP3 and AIM2 inflammasome activation, in turn inducing pyroptosis or IL-1β maturation. This controlled inflammasome activity favors persistence and granulomatous pneumonia. Abbreviations: AIM2, absent in melanoma 2; CCV, *Coxiella*-containing vacuole; DAMPs, damage-associated molecular patterns; ICAM-1, intercellular adhesion molecule 1; IL, interleukin; iNOS, inducible nitric oxide synthase; JNK, c-Jun N-terminal kinase; MCP-1, monocyte chemoattractant protein-1; MyD88, myeloid differentiation primary response 88; NF-κB, nuclear factor kappa-light-chain-enhancer of activated B cells; NLRP3, NOD-, LRR- and pyrin domain-containing protein 3; p38 MAPK, p38 mitogen-activated protein kinase; TLR, Toll-like receptor; TNF-α, tumor necrosis factor alpha; VE-cadherin, vascular endothelial cadherin.

**Figure 2:**
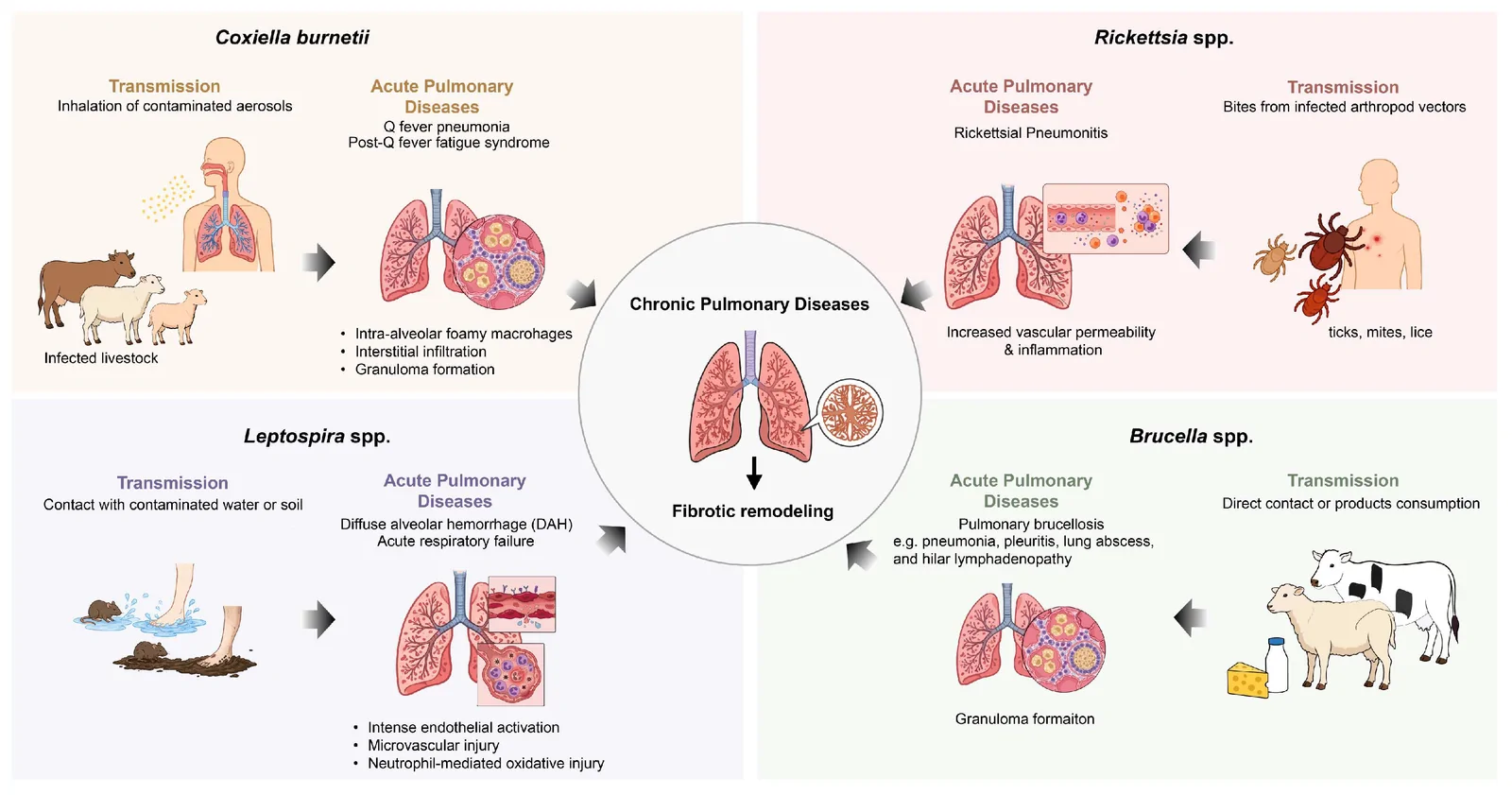
A route from zoonotic infection to pulmonary fibrosis. Each quadrant summarizes the transmission route, acute lung disease, and key pathological features of representative pathogens. *Coxiella burnetii* (**top left**), acquired by breathing in contaminated aerosols from infected livestock, causes Q fever pneumonia marked by foam-like macrophages within the air sacs, interstitial infiltration, and granuloma formation. *Rickettsia* spp. (**top right**), transmitted by arthropod vectors (ticks, mites, lice), cause rickettsial pneumonitis with endothelial injury and increased vascular permeability. *Leptospira* spp. (**bottom left**), acquired through contaminated water or soil, cause diffuse alveolar hemorrhage (DAH; bleeding into the air sacs) and acute respiratory failure via endothelial activation, microvascular injury, and neutrophil-mediated oxidative injury. *Brucella* spp. (**bottom right**), transmitted by contact with infected animals or unpasteurized products, cause pulmonary brucellosis (pneumonia, pleuritis, lung abscess, hilar lymphadenopathy) with granuloma formation. Although the four pathogens belong to very different bacterial groups, they share intracellular or endovascular persistence and convergent profibrotic signaling (**center**), hypothesized to drive chronic pulmonary disease and fibrotic remodeling.

**Figure 3:**
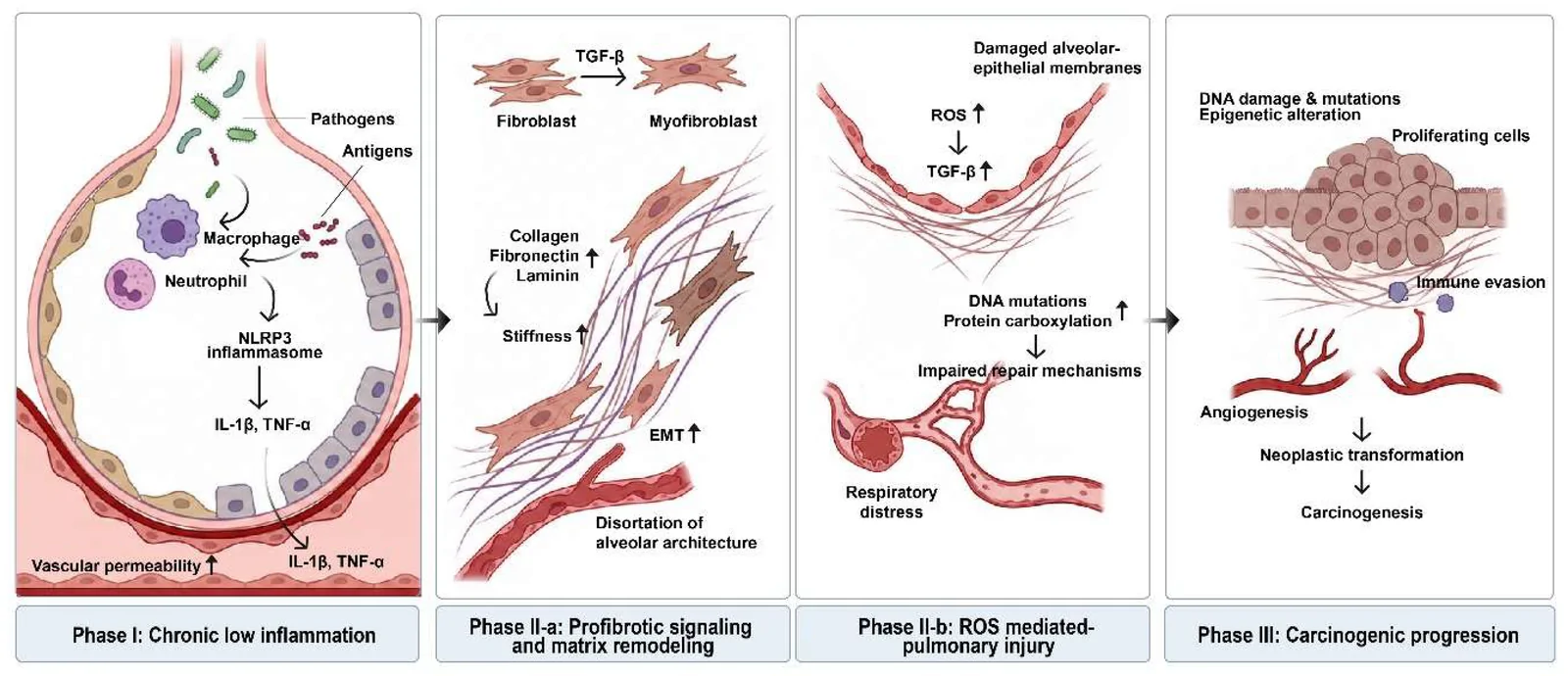
Mechanistic cascade linking zoonotic infection to carcinogenesis. The figure illustrates three sequential and interconnected phases driving the pathological continuum from initial infection to lung cancer. Phase I represents pathogen-driven chronic inflammation, characterized by NLRP3 inflammasome activation and IL-1β/TNF-α secretion. Phase II-a and b represent TGF-β-driven fibrotic remodeling and ROS-mediated oxidative DNA damage, respectively. Phase III represents the resulting genomically unstable, immune-evasive microenvironment that drives neoplastic transformation. Upward arrows indicate an increase in the corresponding factor or process. ECM, extracellular matrix; EMT, epithelial–mesenchymal transition; IL-1β, interleukin-1β; NLRP3, NOD-, LRR- and pyrin domain-containing protein 3; ROS, reactive oxygen species; TGF-β, transforming growth factor-β; TNF-α, tumor necrosis factor-α.

During the preparation of this manuscript, the authors utilized ChatGPT version GPT-5.5 (OpenAI, San Francisco, CA, USA) and Gemini version 3.1 Pro (Google, Mountain View, CA, USA) to generate initial conceptual pictograms for Figure 1, Figure 2 and Figure 3. These pictograms were then modified, corrected, and finalized by the authors using Adobe Illustrator 2026 version 30.2.1 (Adobe Inc., San Jose, CA, USA) to ensure scientific accuracy. Additionally, these tools assisted with English-language proofreading of the manuscript text. All AI-assisted content was critically reviewed and verified by the authors, who take full responsibility for the accuracy and integrity of the final manuscript.

## 3. Bacterial Zoonoses and Pulmonary Diseases

### 3.1. Coxiella burnetii and Q Fever Pneumonia

*Coxiella burnetii* is an obligate intracellular bacterium and the causative agent of Q fever, historically termed “query fever” due to its initially unidentified etiology. Q fever is a globally distributed zoonosis, primarily transmitted via inhalation of contaminated aerosols from infected livestock, particularly during parturition. Acute Q fever is frequently asymptomatic or self-limiting; symptomatic cases typically present with febrile illness accompanied by retro-orbital headache and fatigue [11,12]. Pulmonary involvement occurs in approximately 15 to 40% of symptomatic cases, with considerable geographic variation [4,13,14]. Q fever pneumonia is characterized by intra-alveolar foamy macrophages, interstitial infiltrates, and granuloma formation, reflecting the organism’s capacity to survive and replicate within the phagolysosomal compartment of alveolar macrophages [4,5].

At the molecular level, C. burnetii is sensed primarily through Toll-like receptor 2 (TLR2) with a lesser contribution from TLR4. MyD88-dependent signaling downstream of these receptors is required for bacterial control specifically in the lung, as shown in TLR2/TLR4/MyD88-deficient murine models of pulmonary infection [15]. Despite this recognition, the pathogen actively disrupts downstream innate immune effector mechanisms through its Type IVB secretion system (T4BSS), a bacterial transporter that delivers effector proteins directly into host cells. One such effector protein, IcaA, inhibits the caspase-11-mediated non-canonical activation of the NLRP3 inflammasome [16,17]. In parallel, T4BSS effectors actively suppress IL-17 target gene expression and IL-17-mediated bactericidal activity within infected macrophages (Figure 1A) [18]. Together, these evasion strategies limit effective bacterial clearance and contribute to the sustained intracellular persistence discussed further below.

Acute Q fever is effectively treated with doxycycline; however, the emergence of antibiotic-resistant strains has been increasingly documented and represents a growing clinical concern [19,20]. Approximately 5% of acutely infected individuals progress to chronic Q fever, a more severe and persistent form of the disease with a case fatality rate of around 25% in nationwide cohorts [21,22,23,24]. Chronic pulmonary Q fever manifests as organizing pneumonia or progressive interstitial lung disease. Furthermore, post-Q fever fatigue syndrome characterized by persistent respiratory symptoms, cognitive impairment, and reduced quality of life has been documented in a substantial proportion of survivors following large-scale outbreak investigations [7,25,26]. Collectively, these clinical and epidemiological observations suggest that *C. burnetii* has the potential to establish a state of persistent, low-grade pulmonary inflammation that may not be adequately captured by standard clinical follow-up protocols.

### 3.2. Leptospira spp. and Pulmonary Hemorrhage Syndrome

*Leptospira* spp. are motile, obligate aerobic spirochetes characterized by a cytoplasmic membrane and cell wall enclosed within a porin-containing outer membrane [27,28]. Leptospirosis is one of the most widespread zoonotic diseases globally, with an estimated incidence exceeding one million severe cases and approximately 60,000 deaths annually [29]. Transmission occurs predominantly through direct contact with water or soil contaminated with the urine of infected reservoir animals, and is particularly prevalent in tropical and subtropical regions, where flooding events dramatically amplify exposure risk [30,31]. While many cases present as a self-limiting febrile illness with mild hepatic dysfunction, a severe form called Weil’s disease is characterized by multi-organ failure involving liver, lungs, and kidneys [6,32,33]. Pulmonary involvement occurs in approximately 20% to 70% of severe leptospirosis cases [34], which carries a case fatality rate as high as 74% in some cohorts [32]. Leptospiral pulmonary hemorrhage syndrome (LPHS) represents the most life-threatening pulmonary complication, manifesting as diffuse alveolar hemorrhage (DAH) and acute respiratory failure. *Leptospira* outer membrane components, particularly leptospiral lipopolysaccharide (LPS) and outer membrane proteins (OMPs), trigger intense endothelial activation and microvascular injury within the pulmonary circulation, resulting in DAH [6,35].

Mechanistically, leptospiral LPS is structurally atypical and is recognized predominantly via TLR2 rather than the canonical TLR4-LPS axis in humans, while recognized via both TLR2 and TLR4 in a murine model [36]. It activates pro-inflammatory cytokines such as IL-6 and TNF-α via the NF-κB pathway in macrophages [37,38]. Particularly, the LPS of *L. interrogans* strain 56606v was identified as signaling almost exclusively through TLR4, and TLR4-deficient mice showed a higher bacterial burden yet markedly milder pulmonary and subcutaneous hemorrhage than wild-type mice. Hemorrhage severity in these TLR4-deficient mice closely tracked the time course of iNOS induction. This indicates that TLR4/iNOS-driven inflammatory injury, rather than bacterial burden itself, is the proximate mechanism of pulmonary hemorrhage [39]. In a human endothelial cell infection model, *Leptospira interrogans* disrupts adherens junction architecture, with reduced and mislocalized VE-cadherin-associated junctional proteins accompanying increased ICAM-1 surface expression (Figure 1B) [40]. Although prospective long-term data on pulmonary function in leptospirosis survivors remain limited, one prospective cohort study found that approximately 67% of survivors still have persistent restrictive spirometry abnormalities at day 28 [8]. Furthermore, neutrophil-mediated oxidative injury within the alveolar compartment has been proposed as a contributor to secondary airway and parenchymal damage, potentially predisposing survivors to progressive pulmonary dysfunction [8,41].

### 3.3. Rickettsia spp. and Rickettsial Pneumonitis

*Rickettsiae* are obligate intracellular, Gram-negative bacteria transmitted primarily through the bites of infected arthropod vectors, including ticks, mites, and lice [42,43]. Pathogenic *Rickettsia* spp. are broadly classified into two major groups: the spotted fever group (SFG) and the typhus group (TG) [44]. The SFG includes *R. rickettsii*, *R. conorii*, *R. africae*, and *R. australis*, whereas the TG comprises *R. typhi* and *R. prowazekii.* Rickettsial infections occur globally, with species distribution determined by vector ecology, reservoir host, and climate [43]. Pulmonary involvement in rickettsial disease is well documented; pneumonitis occurs in approximately 10% to 40% of cases, including Rocky Mountain spotted fever (RMSF) caused by *R. rickettsii* and Mediterranean spotted fever (MSF) caused by *R. conorii* [45]. Among hospitalized patients with severe RMSF, a case fatality rate of 21% has been reported, particularly when diagnosis and doxycycline initiation are delayed [46]. Following introduction via arthropod bite, rickettsiae disseminate through the lymphatic and circulatory systems to the systemic and pulmonary circulations. High affinity of *Rickettsia* spp. for endothelial cells results in increased vascular permeability and inflammation. Although long-term neurological sequelae including paraparesis, peripheral neuropathy, and motor dysfunctions were reported in severe cases, chronic pulmonary outcomes have not been systematically investigated [46,47]. Theoretically, endotheliotropic infection drives sustained pro-inflammatory signaling cascades, leading to fibrotic remodeling in the pulmonary system [48].

*Rickettsia* spp. invade host endothelial cells through a receptor-ligand interaction distinct from classical pattern-recognition mechanisms. The rickettsial outer membrane protein B (rOmpB) engages Ku70, a surface invasion receptor, triggering actin recruitment and internalization of the bacterium [49]. In vitro infection of human vascular endothelial cells demonstrated that *R. rickettsii* activates NF-κB and p38 MAP kinase, driving secretion of IL-8 and monocyte chemoattractant protein-1 (MCP-1) [50]. Rickettsial infection induces tyrosine phosphorylation of VE-cadherin, weakening adherens junction and increasing paracellular permeability in vitro [51]. NF-κB activation in this system is anti-apoptotic; blocking NF-κB in infected endothelial cells triggers rapid host cell apoptosis. This indicates that sustained NF-κB signaling is required for endothelial cell survival, which permits ongoing rickettsial replication and cell-to-cell spread (Figure 1C) [52]. This is associated with connective tissue disease-associated interstitial lung diseases (CTD-ILD), in which perivascular inflammation of comparable histological character is well established as a driver of progressive fibrosis [48,53,54]. Although direct evidence from rickettsial disease is currently absent, the biological plausibility, combined with the near-complete absence of prospective follow-up data, represents a significant knowledge gap identified in this review.

### 3.4. Brucella spp. and Pulmonary Brucellosis

*Brucella* spp. are Gram-negative, facultative intracellular coccobacilli transmitted to humans via direct contact with infected animals or consumption of unpasteurized animal products. *B. melitensis*, *B. abortus*, and *B. suis* are the most well-recognized clinically relevant species [55,56,57]. Pulmonary involvement in *Brucella* spp. is reported in up to 20% of cases, with considerable geographic variation. The majority of pulmonary presentations are mild, manifesting as a dry cough without significant respiratory signs. Severe pulmonary manifestations such as pneumonia, pleuritis, lung abscess, and hilar lymphadenopathy occur in approximately 1% to 5% of cases [58,59,60]. Overall mortality from brucellosis is low, with case fatality rates estimated at approximately 1% to 2%, primarily due to endocarditis [55]. Chronic brucellosis, arising from antibiotic resistance or inadequate treatment duration, affects approximately 5% to 15% of patients even after standard antibiotic therapy and may persist for months to years [61,62]. It is diagnosed on the basis of persistent clinical manifestations including spondylitis, musculoskeletal pain, depression, or chronic fatigue syndrome (CFS) in conjunction with high immunoglobulin G titers. Notably, *Brucella* DNA has been detected in patients for years after apparent symptomatic resolution [63,64]. This suggests ongoing subclinical persistence and the need for long-term observation. *Brucella* spp. are capable of inducing granuloma formation, structured aggregates of macrophages and other immune effector cells, associated with the pathogenesis of tuberculosis [65].

*Brucella* spp. are recognized through TLR2 and TLR4, which cooperatively drive p38/JNK MAPK and NF-κB activation and downstream TNF-α, IL-6, and IL-12 production in macrophages [66]. In an intranasally infected murine model, TLR2 and TLR4 are required for pulmonary bacterial clearance [66,67]. Beyond surface TLRs, *Brucella*-associated damage-associated molecular patterns (DAMPs) and cytosolic bacterial DNA activate the NLRP3 and AIM2 inflammasomes, respectively. Both converge on caspase-1 activation, driving pyroptosis and IL-1β maturation [68]. Rather than simply suppressing this response, *Brucella* appears to modulate its intensity: flagellin-deficient mutants that evade inflammasome activation paradoxically exhibit a higher bacterial burden yet impaired establishment of chronic infection, suggesting that controlled inflammasome activity is required to sustain the granulomatous, subclinical persistence of chronic brucellosis (Figure 1D) [68]. This offers a molecular explanation for how the organism maintains granulomatous subclinical persistence.

Given that chronic granulomatous inflammation is well established as a driver of fibrotic remodeling in other pulmonary diseases, the possibility that chronic pulmonary brucellosis may leave a fibrotic legacy remains, although direct evidence from brucellosis cohort cases has not yet been reported. The acute pulmonary involvement, case fatality rates, and chronic progression rates of the four pathogens are summarized in Table 2. The transmission routes of these four zoonotic bacterial pathogens are compared in Figure 2.

### 3.5. Shared Features and the Rationale for a Unified Framework

Despite their phylogenetic diversity—spanning obligate intracellular bacteria (*Coxiella burnetii*, *Rickettsia* spp.), facultative intracellular coccobacilli (*Brucella* spp.), and a motile aerobic spirochete (*Leptospira* spp.)—the four pathogens reviewed above share several pathologically significant features. These features may support the unifying hypothesis that they can establish sustained persistence within pulmonary tissue beyond the acute pneumonia symptoms, despite differing pulmonary injury mechanisms. As summarized in Table 3, each pathogen preferentially targets either macrophages *(C. burnetii*, *Brucella* spp.) or the vascular endothelium (*Leptospira* spp., *Rickettsia* spp.). However, the strength of documented evidence for chronic pulmonary sequelae varies markedly among the pathogens. Critically, none of the four pathogens has been subjected to systematic prospective investigation of long-term pulmonary outcomes. Thus, the unifying framework proposed here rests on convergent mechanistic tropism rather than on directly demonstrated, comparable clinical endpoints across all four pathogens.

## 4. Molecular Mechanisms from Chronic Inflammation to Fibrotic Remodeling and Malignancy

### 4.1. Chronic Inflammation: IL-1β, TNF-α, and Persistent Immune Activation

Chronic inflammation constitutes a central link between acute pulmonary infections and progressive lung injury. During acute infection, pathogens such as *C. burnetii* or *Leptospira* spp. trigger robust innate immune responses characterized by the recruitment of macrophages and neutrophils [18,69]. For instance, *C. burnetii* invades host macrophages via classic phagocytic mechanisms and matures within a phagolysosome-like compartment known as the *Coxiella*-containing vacuole (CCV). This pathogen is capable of surviving in the respiratory tract for extended periods, aided by transmission through both host and environmental reservoirs [69,70]. Notably, a high interleukin-10 (IL-10) microenvironment within host cells facilitates this prolonged bacterial persistence. IL-10 limits the host immune response to pathogens, thereby mitigating collateral tissue damage. Engagement of the IL-10 receptor complex comprising IL-10R1 and IL-10R2 on monocytes and macrophages activates the JAK/STAT signaling cascade, leading to the suppression of pro-inflammatory mediators [71]. However, this survival strategy fosters persistent antigenic stimulation, which maintains a state of low-grade but sustained inflammation in the lung.

Among the key regulators of this chronic inflammation, interleukin-1 β (IL-1β) plays a pivotal role by amplifying the inflammatory cascade through NLRP3 inflammasome activation and the promotion of neutrophil infiltration [72,73,74]. Elevated IL-1β levels are well-documented in both infectious pneumonias and IPF, highlighting its dual role as an essential host defense mediator and a primary driver of pathogenic tissue remodeling [75,76,77]. Tumor necrosis factor- α (TNF-α) is another critical cytokine produced by activated macrophages and infected endothelial cells [78,79]. By enhancing vascular permeability and leukocyte trafficking, TNF-α exacerbates alveolar injury [80,81]. Notably, prolonged TNF-α signaling promotes fibroblast proliferation and extracellular matrix (ECM) deposition, laying the groundwork for fibrotic remodeling [82,83]. Consequently, persistent immune activation results in a self-perpetuating inflammatory microenvironment. Even following the apparent resolution of acute infection, residual, highly stable pathogenic antigens such as the highly stable lipopolysaccharide of *C. burnetii* or the outer membrane components of *Leptospira*—or sustained immune cell dysregulation can perpetuate cytokine production, thereby maintaining chronic, low-level inflammation. Over time, this unresolved inflammatory milieu not only drives fibrotic changes but also engenders conditions conducive to genomic instability, oxidative stress, and immune evasion, ultimately contributing to a tissue state that may predispose the injured lung to malignancy [84].

### 4.2. TGF-β-Mediated Profibrotic Remodeling

Following chronic inflammatory stimulation, profibrotic pathways become activated, driven predominantly by the upregulation of transforming growth factor-β (TGF-β). Secreted by activated macrophages, damaged epithelial cells, and fibroblasts, TGF-β initiates the canonical SMAD2/3 signaling cascade to induce transcription of profibrotic genes [85,86]. It promotes the differentiation of resident fibroblasts into myofibroblasts—a contractile and highly synthetic phenotype responsible for excessive extracellular matrix (ECM) production, which is a key hallmark of established fibrotic disease [87,88].

Once transformed, myofibroblasts deposit key ECM proteins, including collagen type I, fibronectin, and laminin. While these proteins are essential for tissue repair during acute injury, their uncontrolled accumulation under persistent activation thickens alveolar septa, distorts normal lung architecture, and impairs gas exchange, which are defining histological features of pulmonary fibrosis [89,90,91]. For instance, in the context of *Leptospira* spp. infection, diffuse alveolar hemorrhage creates a TGF-β-enriched microenvironment that may prime this fibroblast activation cascade in surviving patients [92]. As progressive collagen deposition and fiber crosslinking occur, tissue stiffness increases, establishing a feed-forward mechanobiological loop. This stiffness activates mechanotransducive signaling cascades through integrin-ECM focal adhesion [93,94]. Key transducer proteins including focal adhesion kinase (FAK), PI3K, RAS, RhoA, and Yes-associated protein (YAP) transmit these mechanical signals to the nucleus, activating proliferative and survival pathways [95]. This mechanobiological cycle locks the lung into progressive fibrosis despite pathogen clearance, indicating that pulmonary sequelae may be able to persist long after the acute infectious episode has resolved.

Beyond these structural changes, this fibrotic signaling reshapes the lung microenvironment to favor malignant transformation. Sustained elevation of TGF-β contributes to epithelial–mesenchymal transition (EMT) [96,97], promotes pathological angiogenesis via vascular endothelial growth factor (VEGF) and platelet-derived growth factor (PDGF) [98], and suppresses local antitumor immunity through regulatory T cell induction and programmed death-ligand 1 (PD-L1) upregulation [99]. Consequently, chronic fibrotic remodeling not only scars the lung but sets the stage for carcinogenesis—bridging infection-induced injury to long-term oncogenic outcomes.

### 4.3. ROS-Mediated Oxidative Stress

Alongside cytokine-driven inflammation, oxidative stress represents a critical, yet often underappreciated, mechanism linking pathogen-mediated infections to chronic pulmonary pathology. During acute infection, professional phagocytes including macrophages and neutrophils generate reactive oxygen species (ROS) as a conserved antimicrobial defense [100,101]. However, this ROS-generating machinery shifts its role from protective to destructive when infection persists or fails to resolve completely.

Intracellular pathogens, such as *Coxiella burnetii* and *Rickettsia* spp., are particularly adept at sustaining oxidative stress within host cells [102,103,104]. By subverting phagosomal maturation and exploiting host mitochondrial function, these organisms create an environment that promotes sustained superoxide and hydrogen peroxide generation [69,105]. This oxidative milieu indiscriminately damages cellular macromolecules: (1) lipid peroxidation disrupts alveolar epithelial membranes; (2) oxidative DNA adducts, particularly 8-oxoguanine, accumulate in type II pneumocytes and alveolar macrophages; and (3) protein carbonylation impairs the function of antioxidant enzymes [106,107,108,109].

At the tissue level, ROS directly activate latent TGF-β, amplifying the profibrotic signaling cascade described above. Furthermore, oxidative stress upregulates the transcription factor NF-κB, sustaining the production of pro-inflammatory cytokines including IL-1β and TNF-α, thereby creating a self-reinforcing loop between inflammation and oxidative damage [110,111]. Accumulation of oxidative DNA lesions in progenitor and stem cells of the lung epithelium constitutes a potent mutagenic substrate. Critically, the resulting genomic landscape of this chronically inflamed, fibrotic lung tissue is inherently mutagenic. For instance, the mutational signature of lung cancer arising in the context of pulmonary fibrosis (LC-IPF) distinctly differs from that of smoking-related lung cancer. Particularly, apolipoprotein B mRNA editing enzyme, catalytic polypeptide (APOBEC)-related mutagenesis, characterized by a predominance of C>T somatic transitions, is a major contributor to LC-IPF. This pattern is mechanistically consistent with sustained cytidine deaminase activity driven by the chronically inflamed and oxidatively stressed epithelium, rather than by tobacco carcinogen exposure [112]. Ultimately, by combining profound oxidative DNA damage with impaired repair mechanisms, this environment fosters the genomic instability necessary for malignant transformation—directly bridging the infectious microenvironment to carcinogenesis [111,113].

### 4.4. Epithelial–Mesenchymal Transition (EMT)

Epithelial–mesenchymal transition (EMT) is a fundamental cellular reprogramming process whereby fully differentiated epithelial cells lose their characteristic polarity and intercellular junctions and acquire a motile, mesenchymal phenotype [114]. Originally described in the context of embryogenesis and wound healing, EMT has emerged as a central mechanism in both pulmonary fibrosis and lung carcinogenesis, representing a critical convergence point at which chronic, infection-induced injury transitions into irreversible structural remodeling [115,116].

In the context of persistent infection and chronic inflammation, EMT is primarily driven by sustained TGF-β signaling acting through the canonical SMAD2/3 pathway [117,118]. Activated SMAD complexes translocate to the nucleus and suppress epithelial fate genes—most notably E-cadherin, whose loss is the defining molecular event of EMT—while simultaneously upregulating mesenchymal markers including vimentin, N-cadherin, and α-smooth muscle actin (α-SMA) [114]. The result is a population of cells capable of invading the extracellular matrix, evading anoikis, and producing abundant collagen, effectively functioning as de facto myofibroblasts that perpetuate fibrotic remodeling [119].

Several non-canonical pathways activated during pathogen-mediated infection further potentiate EMT. ROS-induced activation of the Wnt/β-catenin pathway, as well as Notch signaling triggered by injured endothelial cells, synergizes with TGF-β to enhance and stabilize the mesenchymal phenotype [120,121,122]. For instance, *Leptospira*-associated diffuse alveolar hemorrhage generates a TGF-β and ROS-enriched microenvironment that maximally drives EMT in alveolar type II cells [123,124].

From an oncological perspective, EMT confers properties directly relevant to malignant transformation [125]. Cells undergoing EMT exhibit stem cell-like characteristics, including resistance to apoptosis, enhanced self-renewal capacity, and upregulation of drug efflux transporters [126,127]. Moreover, partial EMT states, which are a hybrid of epithelial and mesenchymal traits, are increasingly recognized as the most tumorigenic, as they combine proliferative capacity with invasive potential [128,129]. Thus, by driving repeated cycles of partial EMT in chronically injured lung epithelium, pathogens may contribute not only to fibrosis but to the emergence of transformed, premalignant cell populations capable of initiating lung carcinogenesis.

### 4.5. From Fibrosis to Malignancy: The Oncogenic Microenvironment of the Scarred Lung

The association between chronic pulmonary fibrosis and lung cancer is supported by substantial mechanistic and epidemiological evidence, although the precise causal pathways remain incompletely understood. While pulmonary fibrosis is a well-established risk factor for lung cancer, the extension of this relationship to post-infectious fibrosis remains hypothetical and requires direct validation in longitudinal human studies. Patients with pulmonary fibrosis carry a significantly elevated risk of developing primary lung cancer, with adjusted odds ratios of 2.58 (95% CI: 2.56–2.60) reported in large-scale analyses exceeding one million patients [130,131]. Kaplan–Meier analyses further demonstrate that approximately 15% of patients with pulmonary fibrosis develop primary lung cancer within 15 years of initial diagnosis, with the highest risk concentrated within the first five years [130]. Consistent with this early concentration of risk, an independent idiopathic pulmonary fibrosis cohort reported a 5-year cumulative lung cancer incidence of 15.4%, underscoring that a substantial proportion of cases arise within the first few years after diagnosis [132]. These figures establish pulmonary fibrosis as an independent oncogenic risk factor in its own right—and, by extension, raise the possibility that post-infectious fibrosis of any etiology may carry analogous carcinogenic potential. This concept is further supported by the long-recognized phenomenon of tuberculosis-associated scar cancer [133], suggesting that chronic infection-related fibrotic scars may represent a broader oncogenic niche beyond zoonotic infections.

At least four convergent mechanisms explain this elevated risk. First, fibrotic ECM remodeling fundamentally alters the biomechanical properties of the lung. Second, TGF-β simultaneously functions as a potent immunosuppressant and protumorigenic signal. Third, the genomic landscape of fibrotic lung tissue is inherently mutagenic due to chronic oxidative stress. Lastly, IPF itself meets all five criteria for classification as a precancerous condition established by the National Cancer Institute (Figure 3). Notably, it is histologically and molecularly distinct from normal lung tissue and shares oncogenic features without itself being malignant [131]. This formal precancerous designation is not merely semantic—it has direct implications for surveillance policy, supporting the case for structured oncological monitoring in patients with established post-infectious fibrosis.

The histological distribution of fibrosis-associated lung cancer further reflects the underlying pathological geography. Tumors tend to arise in the lower lobes, in peripheral distributions that mirror the honeycombing pattern of fibrosis, and squamous cell carcinoma predominates—in contrast to the adenocarcinoma predominance seen in the general lung cancer population [131]. When adenocarcinoma does arise in the fibrotic lung, it frequently develops at the fibrotic margin, suggesting that the interface between scarred and relatively preserved tissue constitutes a zone of particular oncogenic vulnerability [134]. These spatial patterns are entirely consistent with the hypothesis that post-infectious fibrosis, whatever its etiology, creates localized niches of genomic instability, aberrant repair signaling, and immune suppression that collectively lower the threshold for malignant transformation.

## 5. Clinical and Translational Implications

### 5.1. Follow-Up of Acute Infection Survivors

Current clinical practice for zoonotic infections such as Q fever, leptospirosis, and rickettsiosis is largely oriented toward the resolution of acute illness, with little systematic attention to long-term pulmonary outcomes. This approach is increasingly difficult to justify in light of accumulating evidence that chronic respiratory sequelae may persist well beyond apparent clinical recovery [117]. Given the significantly increased risk of lung cancer associated with pulmonary fibrosis—especially within the first five years of diagnosis—surveillance strategies may need to be reevaluated in future studies to determine whether early screening provides clinical benefits in this population during this period. Translating this principle to the context of zoonotic infections, we propose that patients who have experienced severe pulmonary involvement during acute infection—including those with documented interstitial pneumonia, diffuse alveolar hemorrhage, or prolonged respiratory dysfunction—should be evaluated in future long-term prospective studies [8]. Potential follow-up strategies that merit evaluation in these studies may include serial pulmonary function testing, radiologic assessments, and biomarker-based risk stratification for selected high-risk survivors.

### 5.2. Cancer Screening and Surveillance: Toward an Expanded Framework

Existing lung cancer screening guidelines, including those updated by the American Cancer Society, primarily focus on high-risk smokers and do not currently consider post-infectious pulmonary fibrosis as an independent indication for surveillance. While the clinical utility of expanding screening to this population has not yet been established, future studies should assess whether a history of severe zoonotic infection with pulmonary involvement serves as an additional risk factor for long-term pulmonary fibrosis and subsequent malignancy [135].

Should future studies confirm that a history of severe zoonotic bacterial infection is an independent risk factor, any expansion of screening eligibility would need to be weighed against potential harms. For instance, false-positive rates in the National Lung Screening Trial ranged from approximately 24% at baseline to 16–27% in subsequent annual screening rounds [136]. Similarly, low-dose CT screening carries an overdiagnosis rate of approximately 18% (95% CI: 0–36%) [137]. While current evidence does not support the notion of long-term psychological harm from screening, there is documented short-term anxiety and distress experienced by patients [137]. Given these considerations, the surveillance proposed in this review is intended to generate hypotheses for future risk-stratified, prospective investigation, not to recommend immediate changes to clinical practice.

### 5.3. Translational Research: Antifibrotic and Antineoplastic Drug Accessibility

The mechanistic overlap between pulmonary fibrosis and lung cancer opens a compelling therapeutic opportunity that remains largely unexplored in the context of post-infectious lung disease. The antifibrotic drugs nintedanib and pirfenidone share several molecular, pathophysiological, and clinical aspects with lung cancer treatment, and the common pathophysiological backgrounds of IPF and lung cancer make possible the mutual or combined use of antifibrotic and antineoplastic drugs to treat these highly lethal diseases [131]. Nintedanib, in particular, is already approved for use in both IPF and advanced non-small cell lung cancer, providing a precedent for dual-indication deployment that could be directly relevant to patients with post-infectious fibrosis who subsequently develop malignancy [138]. Although the translational potential of these agents is promising, both drugs are associated with adverse effects and practical limitations that may complicate their clinical application [139]. As with the screening strategies discussed in Section 5.2, these adverse effects must be weighed against a therapeutic benefit that, in the context of post-infectious pulmonary fibrosis, is still entirely theoretical rather than clinically demonstrated.

Future studies should evaluate whether repurposed antifibrotic or anti-inflammatory agents can attenuate post-infectious fibrotic signaling and interrupt progression toward a carcinogenic microenvironment. In addition, clinical trials of antifibrotic therapies should consider post-infectious etiologies as distinct enrollment strata rather than focusing exclusively on idiopathic disease [140]. Ultimately, translating the mechanistic framework proposed in this review into clinical practice will require interdisciplinary collaboration integrating pulmonary medicine, molecular pathology, infectious disease biology, and oncology. Continued longitudinal investigation of survivors beyond the acute phase will be essential to determine whether intervention during the fibrotic stage can modify the subsequent risk of malignant transformation [141].

## 6. Evidence Gaps and Future Directions

### 6.1. The Absence of Long-Term Cohort Data

The most fundamental obstacle to establishing causal relationships in this field is the near-complete absence of prospective longitudinal studies tracking pulmonary outcomes in survivors of zoonotic infections. To date, the majority of available evidence derives from case reports, small retrospective case series, and mechanistic studies conducted in the context of idiopathic pulmonary fibrosis. Consequently, the specific contribution of *Coxiella burnetii*, *Leptospira* spp., *Rickettsia* spp., and *Brucella* spp. to long-term pulmonary remodeling has not been systematically investigated in well-characterized survivor cohorts.

Future longitudinal studies should incorporate serial pulmonary function testing, high-resolution CT imaging, and biomarker profiling to determine whether persistent infection-associated inflammation contributes to progressive fibrosis and subsequent malignancy. For example, serial assessments performed at 6, 12, 24, and 60 months post-infection would allow both early subclinical change and late fibrotic progression. Identifying genetic, serum, and other biomarkers shared among patients with progressive pulmonary fibrosis, regardless of etiology, is an emerging priority, and incorporating post-infectious etiologies into such studies would substantially broaden our understanding of fibrosis heterogeneity [142].

### 6.2. The Need for Experimental Animal and Organoid Models

Mechanistic understanding of the proposed fibrosis-to-malignancy continuum is further constrained by the absence of experimental models that recapitulate this pathological trajectory in the context of zoonotic bacterial infection. Existing murine models of *C. burnetii* infection, while well established for studying acute immunopathology [11], have not been extended to examine long-term pulmonary remodeling outcomes. In the case of leptospirosis, infection outcomes considerably vary, and existing models do not consistently recapitulate the severity and pulmonary hemorrhagic phenotype observed in human disease [143].

Beyond pathogen-specific models, pulmonary fibrosis research largely depends on the bleomycin-induced lung injury model, which serves as the standard preclinical platform for studying fibrogenesis and antifibrotic therapy [144]. Applying this platform to pathogen-primed inflammatory states, such as by combining a sublethal pathogen challenge with bleomycin or mechanical injury, could more closely mimic infection-associated lung injury. Currently, only a single preclinical model exists that recapitulates the molecular pathogenesis of pulmonary fibrosis-associated lung cancer [145], highlighting the scarcity of experimental platforms capable of capturing the complete trajectory from fibrosis to malignancy proposed in this review.

Complementary to animal models, organoid systems derived from human airway epithelium provide valuable resources for studying pathogen–epithelium interactions and infection-induced fibrotic responses, while reducing reliance on in vivo systems that do not fully replicate human disease. Similar organoid platforms have already been used to model chronic intracellular bacterial infections and their downstream fibrotic consequences in the context of *Mycobacterium tuberculosis* [146]. This suggests a feasible template for investigating the pulmonary consequences of zoonotic bacterial infection at the epithelial level.

### 6.3. Research Gaps and Future Directions

Despite the compelling mechanistic rationale outlined in this review, evidence linking zoonotic infections to chronic pulmonary fibrosis and lung cancer remains limited. Many of the proposed mechanisms are based more on biological plausibility than direct clinical evidence. Therefore, future studies are needed to validate the fibrosis-to-malignancy continuum proposed in this review through epidemiological, experimental, and translational research.

## 7. Conclusions

Zoonotic bacterial infections have traditionally been viewed primarily as acute febrile illnesses, often considered resolved once the acute phase subsides. This review proposes a conceptual framework that extends beyond the conventional acute infection model. By synthesizing available evidence on the pulmonary pathobiology of *Coxiella burnetii*, *Leptospira* spp., *Rickettsia* spp., and *Brucella* spp., we outline a mechanistically coherent and clinically plausible pathway through which these pathogens may initiate a cascade that extends far beyond the acute episode. This pathway includes alveolar injury, persistent immune activation, oxidative stress, fibrotic remodeling, epithelial–mesenchymal transition, and ultimately malignant transformation.

The four mechanistic pillars reviewed here—chronic inflammation driven by IL-1β and TNF-α, profibrotic signaling via TGF-β and ECM remodeling, ROS-mediated genomic instability, and EMT-facilitated oncogenic reprogramming—are not unique to idiopathic pulmonary fibrosis. Rather, they represent shared biological responses to sustained lung injury of any etiology. This review argues that zoonotic infections, due to their ability to induce persistent intracellular infection, diffuse alveolar hemorrhage, and chronic immune dysregulation, may engage these pathways in a manner that could contribute to fibrotic remodeling and create a microenvironment conducive to malignant transformation over the course of several years.

This perspective has immediate implications for clinical practice. At institutions managing patients with complex interstitial lung disease and lung cancer, obtaining a detailed exposure history—such as residence in or travel to regions endemic to Q fever, leptospirosis, or rickettsial disease, as well as occupational or environmental contact with arthropod vectors—should become a routine part of the respiratory evaluation. The possibility that a patient’s fibrotic lung disease or peripheral lung cancer may have arisen from a prior, incompletely recognized zoonotic infection is currently neither systematically considered nor investigated. Incorporating this possibility into clinical reasoning and diagnostic workup represents a low-cost, immediately actionable change in practice.

We acknowledge that the evidence presented in this review is primarily inferential. Direct epidemiological data establishing zoonotic infections as independent risk factors for pulmonary fibrosis or lung cancer are currently lacking, and the proposed association should be viewed as a biologically plausible hypothesis rather than a demonstrated causal relationship. Similarly, the mechanistic pathways described here, while supported by established molecular biology, require validation through well-designed longitudinal studies and appropriate experimental animal and organoid models.

Ultimately, this review offers a conceptual framework that connects two fields traditionally investigated separately: zoonotic infectious diseases and chronic pulmonary pathology. By integrating current knowledge of infection biology, fibrosis, and carcinogenesis, we propose a unified hypothesis that may guide future mechanistic, translational, and clinical research. Whether post-infectious pulmonary fibrosis constitutes a distinct pathway within the fibrosis-to-malignancy continuum remains to be determined, but this framework provides a foundation for future experimental and longitudinal investigations.

## Figures and Tables

**Table 1:** Overview of representative zoonotic bacterial pathogens and their major animal reservoirs.

Genus	Pathogen	Major Animal Reservoirs
*Coxiella*	*C*. *burnetii*	Livestock, Pets, and Ticks
*Leptospira*	*L. kirschneri*	Mice, Bats, Raccoon
	*L. borgpetersenii*	Mice
	*L. interrogans*	Livestock, Rats
*Rickettsia*	*R. prowazekii*	Lice, Squirrels
	*R. typhi*	Rodents, Fleas
	*R. rickettsii*	Ticks
	*R. conorii*	Dogs, Ticks
	*R. africae*	Ticks
*Brucella*	*B. melitensis*	Sheep, Goats
	*B. abortus*	Cattle
	*B. suis*	Pigs

**Table 2:** Epidemiological outcomes of zoonotic bacterial infections.

Pathogen	Acute Pulmonary Involvement	Case Fatality Rate	Chronic Progression Rate
*Coxiella burnetii*	~15–40%	~25% (confirmed chronic Q fever)	~5% progress to chronic Q fever
*Leptospira* spp.	~20–70%	~74% (severe LPHS cases)	~67% persistent restrictive abnormality at day 28
*Rickettsia* spp.	~10–40%	~21% (hospitalized/severe cases)	Not established
*Brucella* spp.	~20%	~1–2% (primarily endocarditis)	~5–15% relapse after standard therapy

**Table 3:** Comparative pulmonary pathophysiology across the four zoonotic bacterial pathogens.

Pathogen	Route of Transmission	Intracellular/ Vascular Tropism	Pulmonary Injury Mechanism	Chronic Pulmonary Sequelae
*Coxiella burnetii*	Inhalation of contaminated aerosols	Intracellular, macrophage-tropic	Granuloma formation	Chronic Q pneumonia
*Leptospira* spp.	Direct infected-urine contact, urine-contaminated fomite contact	Extracellular, endothelium-tropic	Endothelium activation, microvascular injury	Persistent restrictive spirometry abnormality
*Rickettsia* spp.	Bite	Intracellular, endotheliotropic	Endothelial injury, increased vascular permeability	Not systematically investigated
*Brucella* spp.	Direct animal exposure, animal products	Facultative intracellular, macrophage-tropic	Granuloma formation	Not specifically documented

## Data Availability

No new data were created or analyzed in this study. Data sharing is not applicable to this article.

## Abbreviations

The following abbreviations are used in this manuscript:

AIM2	absent in melanoma 2
APOBEC	apolipoprotein B mRNA editing enzyme, catalytic polypeptide
CCV	*Coxiella*-containing vacuole
CFS	chronic fatigue syndrome
CI	confidence interval
CT	computed tomography
CTD-ILD	connective tissue disease-associated interstitial lung diseases
DAH	diffuse alveolar hemorrhage
DAMPs	damage-associated molecular patterns
ECM	extracellular matrix
EMT	epithelial–mesenchymal transition
FAK	focal adhesion kinase
ICAM-1	intercellular adhesion molecule 1
IL-1β	interleukin-1β
IL-6	interleukin-6
IL-8	interleukin-8
IL-10	interleukin-10
IL-12	interleukin-12
IL-17	interleukin-17
iNOS	inducible nitric oxide synthase
IPF	idiopathic pulmonary fibrosis
JNK	c-Jun N-terminal kinase
LC-IPF	lung cancer arising in the context of pulmonary fibrosis
LDCT	low-dose computed tomography
LPHS	Leptospiral pulmonary hemorrhage syndrome
LPS	lipopolysaccharide
MAPK	mitogen-activated protein kinase
MCP-1	monocyte chemoattractant protein-1
MSF	Mediterranean spotted fever
MyD88	myeloid differentiation primary response 88
NF-κB	nuclear factor kappa-light-chain-enhancer of activated B cells
NLRP3	NOD-, LRR-, and pyrin domain-containing protein 3
OMP	outer membrane protein
PDGF	platelet-derived growth factor
PD-L1	programmed death-ligand 1
RMSF	Rocky Mountain spotted fever
ROS	reactive oxygen species
SFG	spotted fever group
TG	typhus group
TGF-β	transforming growth factor-β
TLR	Toll-like receptor
TNF-α	tumor necrosis factor-α
T4BSS	Type IVB secretion system
VEGF	vascular endothelial growth factor
YAP	Yes-associated protein
α-SMA	α-smooth muscle actin
